# Magnetic Properties and Washability of Roasted Suspended Siderite Ores

**DOI:** 10.3390/ma15103582

**Published:** 2022-05-17

**Authors:** Yanxin Chen, Chao Yang, Shaowu Jiu, Bo Zhao, Qiang Song

**Affiliations:** College of Materials Science and Engineering, Xi’an University of Architecture and Technology, Xi’an 710055, China; ycwork@xauat.edu.cn (C.Y.); jiushaowu@xauat.edu.cn (S.J.); 17792518719@xauat.edu.cn (B.Z.); nj@xauat.edu.cn (Q.S.)

**Keywords:** siderite, magnetization roasting, suspended state, magnetic properties, magnetite, magnetic separation

## Abstract

Steel is one of the most important industrial materials, which mainly comes from the smelting of iron ore. In view of the huge steel consumption every year, the exploitation of vast reserves of siderite ores is significant for improving the self-sufficiency rate of iron ore resources and ensuring the strategic security of the iron and steel industries. This paper investigated the influence of temperature, time, and other parameters on the magnetic properties of roasted siderite ores using the method of suspended roasting and analyzed the washability of roasted ores under weak-magnetic-field conditions using the magnetic separation tube experiment. The findings of the study explained the iron phase transformation process, i.e., FeCO_3_ was transformed into Fe_3_O_4_ by suspension magnetization roasting. Furthermore, the saturation magnetization of the roasted ore increased in due time at a constant temperature range of 550–750 °C and a roasting time of less than 5 s. It also increased with increasing temperature and constant time. The roasted ore achieved the best magnetic characteristics after roasting at 750 °C for 5 s. After low-intensity magnetic separation, the iron grade of the concentrate changed to 55.12%, with a recovery rate of 90.34%. The study results provide a reference for the development and application of siderite suspension magnetization roasting technology.

## 1. Introduction

With the continuous development of the economy in the world, the demand for steel, an important industrial material, is also increasing. For example, China’s steel consumption was close to 1 billion tons in 2021, which put great pressure on the iron ore supply. The iron ores in China are characterized by a considerable quantity of low-grade ores, which are difficult to directly use in iron making. To avoid dependence on imported iron ore, the strategic security of the iron and steel industries in China requires the usage of all refractory low-grade iron ores, including siderite ores [1].

The primary method of using siderite is to convert it into magnetite and its further enrichment with iron via magnetic separation. The reaction of siderite under a neutral atmosphere is a two-step process and is achieved via the chemical reaction shown in Equations (1) and (2) [2]:

FeCO_3_ → FeO + CO_2_↑ (1)

3FeO + CO_2_ → Fe_3_O_4_ + CO↑ (2)

Suspension magnetization roasting is a relatively novel method for treating siderite and hematite [3]. This has attracted widespread attention due to its excellent heat and mass transfer efficiency and low roasting energy consumption [4] in comparison to previous magnetization roasting techniques used in shaft furnaces and rotary kilns. Scholars have conducted a series of studies on the suspension magnetization roasting technology used on low-grade refractory iron ores, such as siderite, hematite, and limonite [5,6,7,8,9]. The magnetization roasting conditions of siderite impact the quality of roasting and determine the magnetic separation effect of the roasted ore. Temperature, atmosphere, and time significantly affect the quality of roasted ores during suspension magnetization roasting [10,11]. The iron content and recovery rate of magnetic concentrates are determined mainly by the magnetic properties of the roasted ore, which are closely related to the formation conditions, grain size, and shape [12,13]. Florek [14] and Znamenackova [15] used microwaves to treat iron ore and investigate the various magnetic properties of treated minerals, including magnetic susceptibility and magnetization. Waters [16] used a muffle furnace to heat treat pyrite, and the saturation magnetization properties of the sample were used for magnetic separation with a recovery rate of 94%. At present, there are many studies on the magnetic characteristics and washability of iron minerals in a stacked state. However, as suspended magnetization roasting technology develops, theoretical research on the magnetic properties and washability of roasted ore as a function of roasting parameters in a suspended state is urgently needed to support technological research and development, as well as production practices.

Daxigou siderite ores from Shaanxi Province, China, were used as the source material for this study. In a small suspension roasting system, low-concentration CO produced by fossil fuel combustion was simulated to magnetize siderite. The effect of temperature and time parameters on the magnetic characteristics and washability of roasted siderite ore was investigated using a vibrating sample magnetometer, X-ray diffraction examination, and low-intensity magnetic separation.

## 2. Materials and Methods

### 2.1. Materials

The raw siderite ore was crushed in a jaw crusher (PowerScreen (PSC 60 × 100), Westport, CT, USA) and then ground in a ball mill (φ305 × 305 mm). Finally, the material was sieved using an 80 µm square hole sieve, and the sieved material was thoroughly homogenized. An appropriate quantity of iron ore powder was dried in an oven at 105 °C for 24 h and cooled to room temperature and then packed in bags using a desiccator. The raw materials were analyzed chemically by iron grade, physical phase, and quantitative analysis of the iron phase.

**Figure 1:**
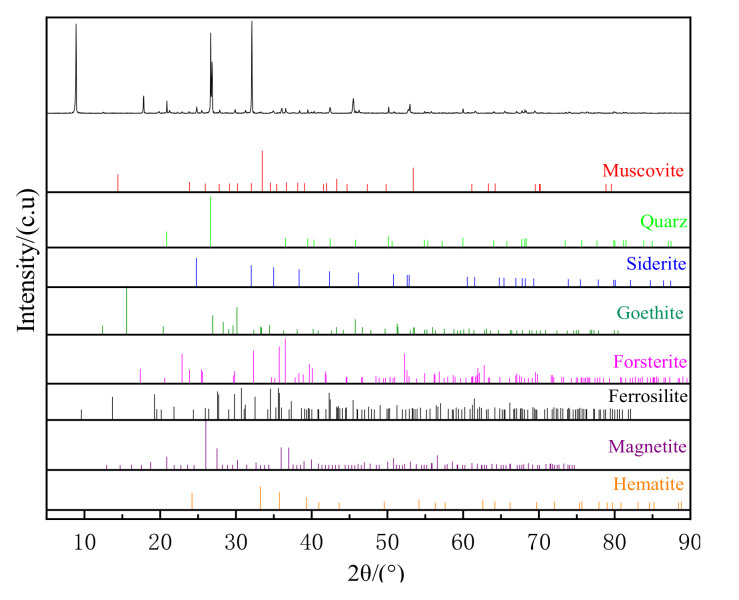
XRD pattern of the sample.

The chemical elemental analysis of the samples was performed using an X-ray fluorescence spectrometer (ARL Perform’X, Thermo Fisher Scientific, Waltham, MA, USA). The X-ray source was a rhodium target, and the results are summarized in Table 1. The results of the siderite iron phase are shown in Table 2. The phase analysis was performed using an X-ray diffraction analyzer (Bruker D8 Advance, Bruker, Billerica, MA, USA), the X-ray source was a copper target, the 2θ step was 0.025°, and the scanning range was 5–90°. The results are depicted in Figure 1.

The X-ray fluorescence analysis results indicated that the most valuable element in the mineral was Fe, with a grade of 22.53%, and the major impurity was SiO_2_ (40.05%). The other impurities were MgO, Al_2_O_3_, and CaO at 1.22%, 13.17%, and 0.31%, respectively, while the harmful elements S and P were only found at levels of 0.14% and 0.04%, respectively. The phase analysis revealed that the iron content of the mineral was composed chiefly of siderite, goethite, and hematite, which accounted for 70.49% of the total iron content in the mineral. X-ray diffraction analysis showed that siderite, goethite, and a small amount of hematite and magnetite were the most prevalent useful minerals, whereas quartz and muscovite were the predominant vein minerals. In addition, the mineral also contained a small amount of forsterite and ferrosilite. Overall, Daxigou siderite ores were a low-grade iron ore.

### 2.2. Methods

#### 2.2.1. Suspension State Magnetization Roasting Test Method

**Figure 2:**
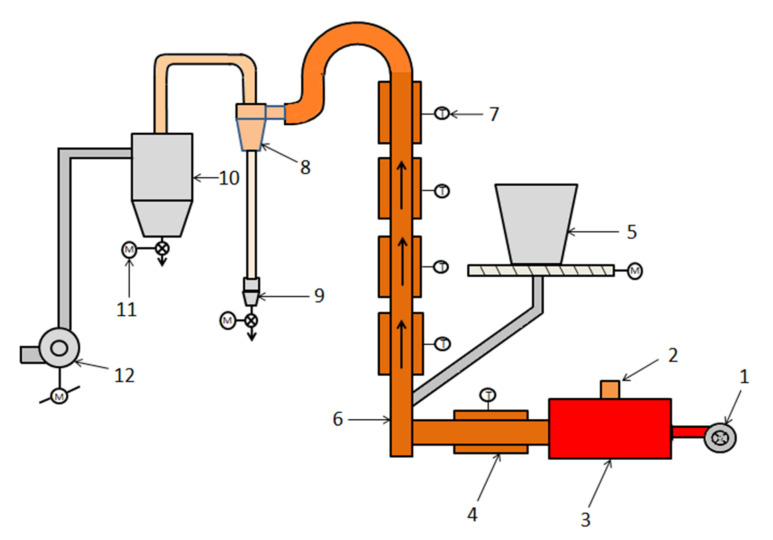
Schematic diagram of the suspended experimental system (1—burner; 2—observation hole; 3—hot blast stove; 4—electric heating furnace; 5—feeding machine; 6—reactor; 7—thermocouple; 8—cyclone; 9—charging basket; 10—dust collector; 11—electric valve; 12—induced draft fan).

The schematic diagram of the suspended-state test setup is shown in Figure 2. The materials were added to the bottom of the reactor by a feeder and drawn up to react by the hot air in the reactor. The reacted materials passed through the cyclone for gas solid separation and then passed into the barrel, which were cooled in the absence of outside air. A hole at the outlet of the hot blast furnace was used to measure the flue gas composition using a portable handheld flue gas analyzer (testo-330, Testo AG, Schwarzwald, Germany).

A fixed dosage rate of 5 kg/h was maintained throughout the test. The outlet temperature of the reaction furnace (measurement point 7 in Figure 2) was regarded as the reaction temperature of the system. This experiment had six temperature points, namely 550 °C, 600 °C, 650 °C, 700 °C, 750 °C, and 800 °C, and the temperature control accuracy was ±5 °C. An electric heating control casing was installed outside the reaction furnace, and the temperature difference compared to the lower part of the furnace body was 20 ± 5 °C. An atmospheric control approach was applied to maintain the CO concentration between 3 and 5% inside the inlet of the reactor (measurement point 6 in Figure 2) by regulating the air–fuel ratio of the hot-blast stove. The materials were easily blocked when the wind speed was too low due to the limited height of the suspension furnace used in the test. On the other hand, when the wind speed was very high, the residence time and roasting of siderite were inadequate. The residence time of materials inside the reactor was around 1 s by adjusting the fan frequency and stabilizing the reactor outlet. Each set of temperature conditions was subjected to five cycles. Given that the suspended state system is capable of instantaneous heat transfer, each cycle executed was comparable to an extended response time of 1 s.

#### 2.2.2. Characterization Method of Roasted Ore

The magnetic properties of the roasted ore were determined by testing the hysteresis curve of the samples to obtain saturation magnetization strength, remanence, and coercivity indices. A vibrating sample magnetometer (LakeShore 7404, LakeShore, Columbus, OH, USA) with a magnetic field range of −50,000 Oe to 50,000 Oe was used for this purpose. The phase of the roasted ore was determined using an X-ray diffraction analysis (as mentioned above).

#### 2.2.3. Magnetic Separation Test

The magnetic separation test was conducted utilizing a magnetic separation tube (RK/CXG-φ50, Rock, Wuhan, China) and the magnetic field strength was 1500 Oe. Roasted ores and concentrates were tested to determine the total iron content.

## 3. Results

### 3.1. Magnetic Properties of Roasted Ores

**Figure 3:**
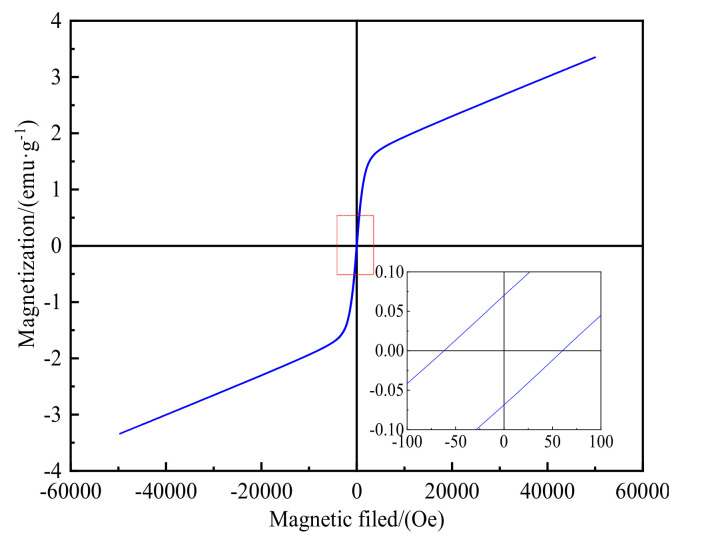
Hysteresis loop of the siderite ore.

The magnetic hysteresis loop of the raw siderite ore is shown in Figure 3, which illustrates that the magnetic hysteresis loop of the raw siderite ore exhibited strong paramagnetic and ferromagnetic characteristics [17], as well as a slight hysteresis phenomenon. When 50,000 Oe was applied, the saturation magnetization strength was 3.35 emu·g^–1^ with a coercivity of 60.31 Oe and a remanent magnetization of 0.07 emu·g^–1^. The saturation magnetization was proportional to the number of magnetic components, evidenced by the increase in the number of magnetic components with an increase in saturation magnetization. As the applied magnetic field intensity was up to 5000 Oe, the magnetic susceptibility decreased sharply, and the hysteresis loop grew linearly. This demonstrates that the siderite raw ore contains both paramagnetic and ferromagnetic materials [18], making it magnetically weak and challenging to beneficiate directly.

**Figure 4:**
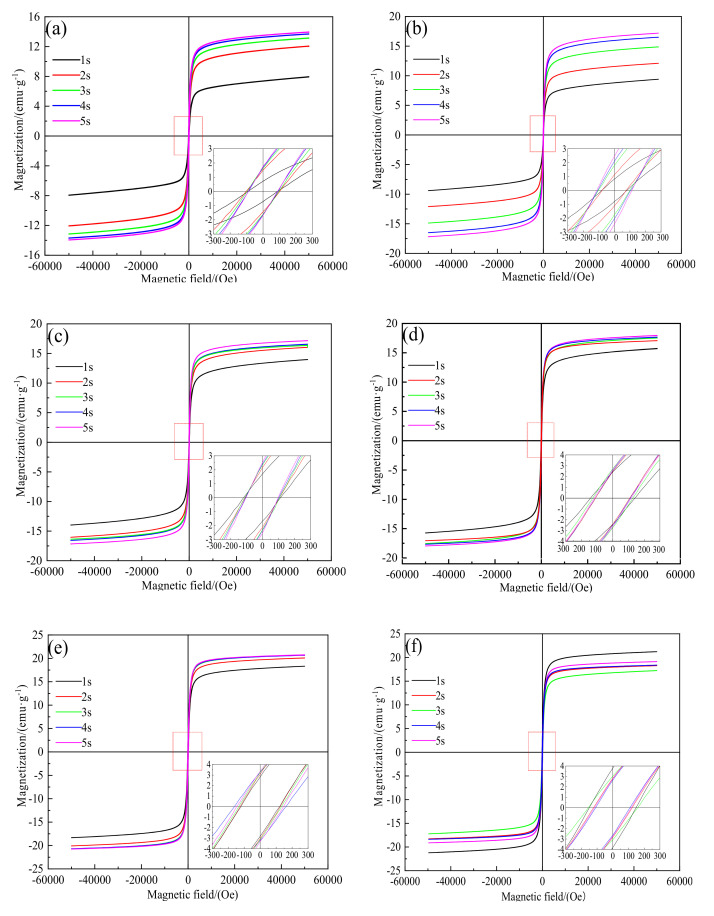
Hysteresis loop of the roasted ore at 550 °C–800 °C. (**a**) Hysteresis loop of the roasted ore at 550 °C. (**b**) Hysteresis loop of the roasted ore at 600 °C. (**c**) Hysteresis loop of the roasted ore at 650 °C. (**d**) Hysteresis loop of the roasted ore at 700 °C. (**e**) Hysteresis loop of the roasted ore at 750 °C. (**f**) Hysteresis loop of the roasted ore at 800 °C.

Figure 4 shows the hysteresis loop for various roasting periods between 550 °C and 800 °C. As shown in Figure 4a, the saturation magnetization of the roasted ore was 7.93 emu·g–1 after 1 s of roasting at 550 °C, which is much higher than 3.35 emu·g–1 of the raw ore, indicating that magnetization occurred. The saturation magnetization and degree of roasted ore magnetization continuously rose with increasing roasting time. The saturation magnetization of the roasted ore reached 13.91 emu·g–1 after 5 s of roasting. When the roasting temperatures were 600 °C, 650 °C, and 700 °C, the trend of the magnetic properties of the roasted ore was the same as when the temperature was 550 °C, which is evident from Figure 4b–d. The saturation magnetization of the roasted ore, on the other hand, steadily increased with the temperature rise. After 5 s of roasting at 600, 650, and 700 °C, the saturation magnetization of the roasted ores was 16.33 emu·g–1, 17.06 emu·g–1, and 17.15 emu·g–1, respectively, indicating that the roasted ores at different temperatures were not completely magnetic below 700 °C for 5 s. The magnetic change in the roasted ore continued to follow the trend of increasing saturation magnetization with time at 750 °C, the hysteresis loops of 3 s, 4 s, and 5 s almost coincide (Figure 4e), with the corresponding saturation magnetizations of 20.09 emu·g–1, 20.69 emu·g–1, and 20.76 emu·g–1, respectively. The corresponding coercivity values were 133.65 Oe, 160.19 Oe, and 146.51 Oe, respectively, and the remanence values were 3.24 emu·g–1, 3.27 emu·g–1, and 3.34 emu·g–1, respectively. This demonstrated that magnetization was completed after 4 s of roasting at 750 °C, which indicates no discernible impact of the prolonged duration. However, when the magnetization temperature increased to 800 °C, the magnetic properties of the roasted ore deviate from the abovementioned rules. As shown in Figure 4f, after roasting for 1 s, the saturation magnetization of the roasted ore showed a maximum value of 21.23 emu·g–1 which did not increase with increasing roasting time though it fluctuated up and down. This shows that the magnetization process can be completed after roasting at 800 °C for 1 s, and increasing the roasting period results in a reduction in the magnetic properties of the roasted ore.

### 3.2. Physical Property Analysis of Roasted Ore

#### 3.2.1. X-ray Diffraction Analysis

**Figure 5:**
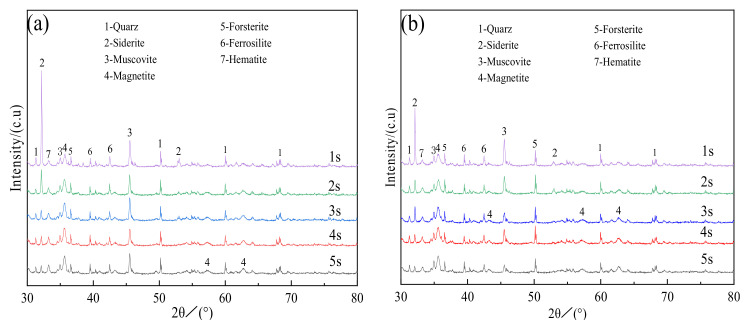

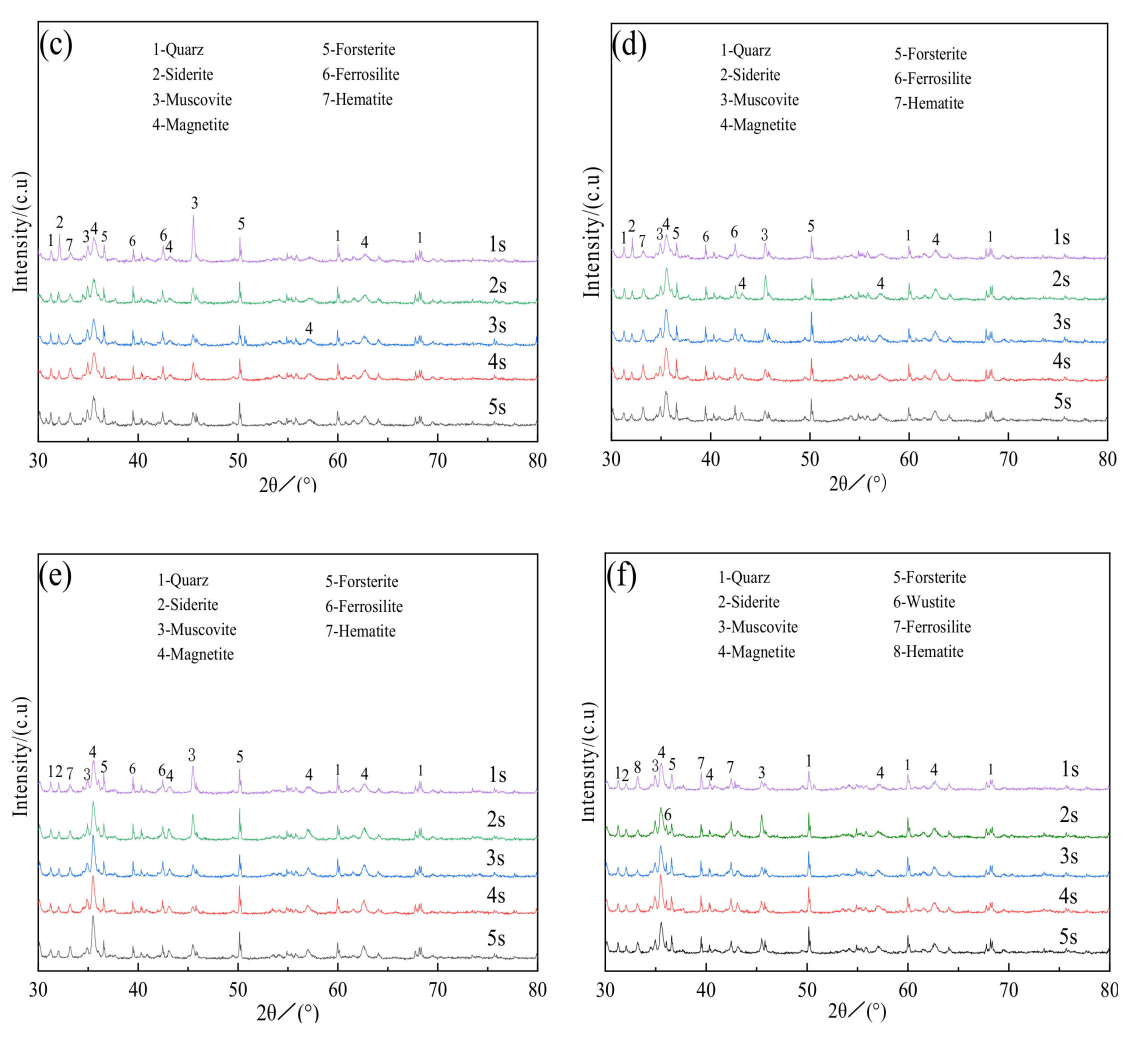
XRD patterns of the roasted ore at 550–800 °C. (**a**) 550 °C; (**b**) 600 °C; (**c**) 650°C; (**d**) 700 °C; (**e**) 750 °C; (**f**) 800 °C.

The XRD patterns of the roasted ore with a roasting time of 1–5 s at 500–800 °C are shown in Figure 5. After roasting at 550 and 600 °C for 1 s, the diffraction peaks of goethite disappeared and the peak of hematite at 2θ = 33.28° became more intense. This is because goethite was transformed into hematite and the diffraction peaks of siderite were still visible, as shown in Figure 5a,b. With an increasing roasting time, the intensity of characteristic diffraction peaks of siderite was reduced, while that of magnetite (2θ = 35.66°) gradually increased, indicating the gradual transformation of siderite to magnetite. Figure 5c–e show that the phase change characteristics of the calcined ore were comparable at 650 °C, 700 °C, and 750 °C over time. After 1 s of roasting at 650 °C and 700 °C, the expected peak of siderite at 2θ = 32.18° reduced significantly, whereas the characteristic peak of siderite at 2θ = 52.97° disappeared completely. However, after 1 s of roasting at 750 °C, the characteristic peak of siderite was nearly totally obscured, suggesting the complete decomposition of siderite. With increasing roasting time, the characteristic diffraction peak of magnetite at 2θ = 35.66° gradually increased, indicating the steady increase in the magnetite content of the roasted ore. The combined analysis in Figure 5c–e shows that after the same roasting time, the intensity of the characteristic diffraction peak of magnetite increased with the rise in the roasting temperature. The X-ray diffraction intensity of magnetite in the roasted ore was the highest after 5 s of magnetization roasting at 750 °C, indicating that this setting provided the best magnetization roasting effect. Figure 5f illustrates that the X-ray diffraction peak of magnetite in the roasted ore obtained at 800 °C, no longer obeyed the rule that the longer the exposure duration, the greater the intensity. By comparing Figure 5e with Figure 5f, it is clear that the X-ray diffraction intensity of magnetite in the roasted ore at 800 °C was significantly lower than at 750 °C, indicating a reduction in magnetite concentration. The characteristic FeO diffraction peak at 2θ = 36.02° was seen in the X-ray diffraction pattern of the calcined ore for 2–5 s as shown in Figure 5f, which resulted in the partial transformation of magnetite into FeO and the reduction in the magnetic properties of the calcined ore at 800°C. The reaction is represented by the following equation [19]:

Fe_3_O_4_ + CO → 3FeO + CO_2_↑ (3)

The trend of phase evolution of iron minerals in the roasted ore was compatible with the magnetic properties described in Section 2.1, indicating that the analysis of the magnetization roasting process of siderite in a suspended state was reasonable.

### 3.3. Low-Intensity Magnetic Separation

**Figure 6:**
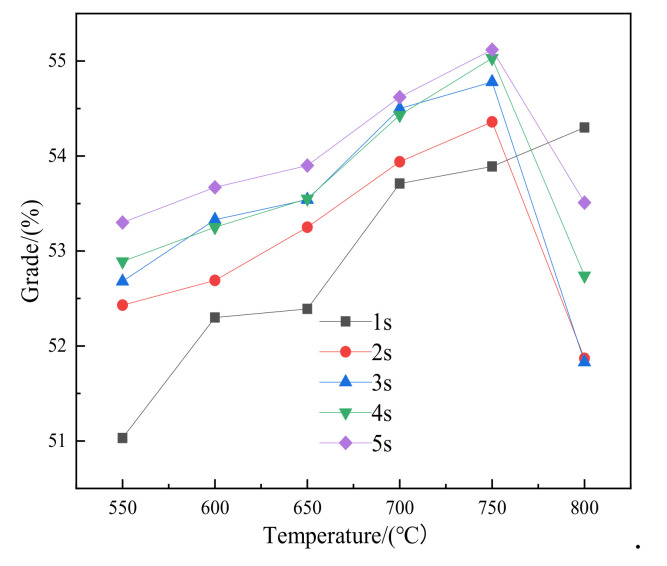
Iron grade diagram of concentrate calcined at 550–800 °C for 1–5 s.

**Figure 7:**
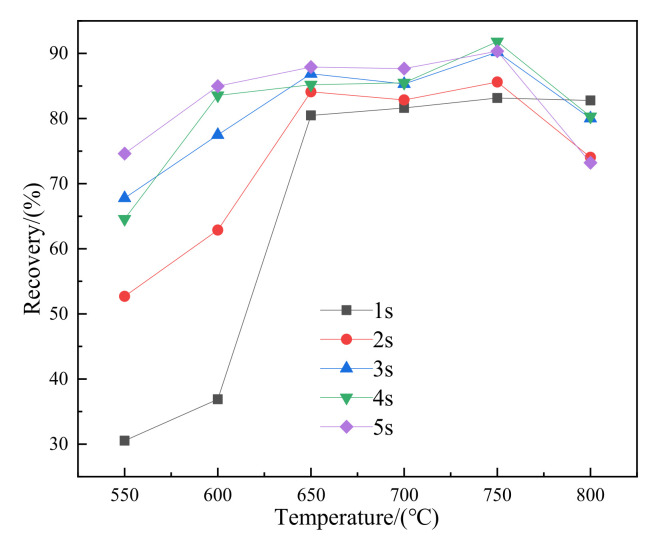
Recovery rate of concentrate calcined at 550–800 °C for 1–5 s.

The iron grade and recovery curves of the magnetic concentrate after roasting at 550–800 °C for 1–5 s are shown in Figure 6 and Figure 7, respectively. Due to the short reaction time, siderite did not completely decompose after roasting at 550 °C for 1s. The iron grade and recovery rate of the concentrate were limited to 51.03% and 30.52%, respectively. The recovery rate of the concentrate increased sharply between 600 °C and 650 °C with an increase in temperature. This can be attributed to the dominance of the decomposition reactions of siderite at temperatures between 490 and 630 °C [20] with higher magnetite concentrations and improved recovery rate at 650 °C. At 800 °C, the iron concentrate grade was 54.3%, while the recovery rate was 82.77%.

The changing trend of iron grade and recovery rate with temperature was the same when the roasting time was extended to 2–5 s. It gradually increased from 550 °C, reached the maximum at 750 °C, and then decreased at 800 °C. After roasting at 550 °C for 5 s, both the iron grade and the recovery rate were greatly improved compared with roasting for 1 s. With the increase in temperature, the iron grade and recovery rate increased, the final iron grade reached the maximum of 55.12% at 750 °C, and the recovery rate reached the maximum of 91.82% at 750 °C. As the temperature continued to rise, the iron grade and recovery rate of the concentrate decreased. In Section 3.2.1, it was proposed that the reason for the decrease of temperature to 800 °C was the presence of FeO in minerals, which was also consistent with the results of magnetometer analysis and X-ray diffraction analysis of vibrating samples. Considering the iron grade and recovery index of the concentrate, the roasted ore obtained after roasting at 750 °C for 5 s had the best quality and the best magnetic separation effect, with the iron grade of 55.12% and the recovery rate of 90.34%.

## 4. Conclusions

(1) Daxigou siderite ores contain paramagnetic and ferromagnetic materials, with weak magnetism making direct beneficiation problematic. Before magnetization roasting using a suspension roasting device, the saturation magnetization of the raw ore was only 3.35 emu·g–1, but after roasting, the maximum magnetization reached 21.23 emu·g–1. Roasting can obviously enhance the magnetism of siderite ores.
(2) When the siderite ore was roasted at 550–750 °C, the saturation magnetization and remanence increased as the roasting time increased. The saturation magnetization of the roasted ore was 20.76 emu·g–1, the coercivity was 146.51 Oe, and the remanence was 3.34 emu·g–1 after 5 s of roasting at 750 °C.
(3) Siderite ores displayed an evident iron phase transformation during magnetization roasting, in which FeCO3 was transformed into Fe3O4, however, the other minerals, such as quartz and muscovite, hardly reacted. When roasting at 800 ℃ for 2 s, Fe3O4 in the roasted ore was excessively restored by CO to FeO, and FeO still existed when the time was 3–5 s, which was unfavorable for magnetic separation.
(4) Before 800 °C, the iron grade and recovery rate of the concentrate obtained by magnetic separation basically increased with the increase in roasting time and temperature. The iron concentrate with an iron grade of 55.12% and recovery rate of 90.34% could be obtained via magnetic separation of the roasted ore obtained after roasting at 750 °C for 5 s. If the temperature continues to rise, the iron grade and recovery rate of the concentrate after magnetic separation will decrease to some extent. On the whole, the best roasting condition of siderite is 750 °C for 5 s.
(5) The vibrating sample magnetometer can provide the basis for magnetic separation. When the minerals reach the saturation magnetization state, the magnetic field required for magnetic separation can be judged by measuring the external magnetic field applied at this time, thus reducing unnecessary energy consumption and improving mineral processing efficiency.

## Figures and Tables

**Table 1:** Chemical element analysis in the study sample (wt.%).

Element	TFe	FeO	SiO2	MgO	CaO	Al2O3	Mn	S	P
Content	22.53	37.76	40.05	1.22	0.31	13.17	0.71	0.14	0.04

**Table 2:** Chemical phase analysis of iron in the study sample (wt.%).

Iron Phase	Fe in Magnetite	Fe in Goethite and Hematite	Fe in Siderite	Fe in Silicate	TOTAL iron
Content	1.33	4.41	11.47	5.32	22.53
Percentage	5.90	19.58	50.91	23.61	100

## Data Availability

The data used to support the findings of this study are available from the corresponding author upon request.
